# Skin Hypopigmentation in Hematology Disorders

**DOI:** 10.3390/hematolrep16020036

**Published:** 2024-06-04

**Authors:** Roberto Mazzetto, Paola Miceli, Alvise Sernicola, Jacopo Tartaglia, Mauro Alaibac

**Affiliations:** Dermatology Unit, Department of Medicine (DIMED), University of Padua, 35121 Padova, Italy; roberto.mazzetto@studenti.unipd.it (R.M.); jacopo.tartaglia@studenti.unipd.it (J.T.); mauro.alaibac@unipd.it (M.A.)

**Keywords:** vitiligo, morphea, syndromic albinism

## Abstract

Hypopigmentation disorders pose significant diagnostic challenges in dermatology, sometimes reflecting underlying hematological conditions. This review explores the clinical presentations related to hypopigmentation in hematological disorders, focusing on vitiligo, morphea, and syndromic albinism. Vitiligo, an autoimmune disorder targeting melanocytes, involves interactions between genetic polymorphisms and immune responses, particularly regarding CD8+ T cells and IFN-γ. Drug-induced vitiligo, notably by immune checkpoint inhibitors and small-molecule targeted anticancer therapies, underscores the importance of immune dysregulation. Morphea, an inflammatory skin disorder, may signal hematological involvement, as seen in deep morphea and post-radiotherapy lesions. Syndromic albinism, linked to various genetic mutations affecting melanin production, often presents with hematologic abnormalities. Treatment approaches focus on targeting the immune pathways specific to the condition, and when that is not possible, managing symptoms. Understanding these dermatological manifestations is crucial for the timely diagnosis and management of hematological disorders.

## 1. Introduction

Hypopigmentation disorders present a challenging spectrum of dermatological conditions, which can arise either as primary dermatopathies or as manifestations of systemic disorders, including hematological conditions. In this article, we aim to explore the main dermatological presentations of hematological interests, focusing on vitiligo, morphea, and albinism. This review provides a concise overview of the pathogenic mechanisms, underlying causes, and management strategies of these dermatological aspects. 

## 2. Vitiligo

### 2.1. Definition and Pathogenesis

Vitiligo is an autoimmune depigmenting skin disorder that causes spots of depigmentation anywhere on the body by targeting melanocytes. It is a multifactorial disease influenced by genetic, immune, and environmental factors, as well as by the use of certain medication. Vitiligo destroys interfollicular epidermal melanocytes, sparing melanocytes residing within immune-privileged hair follicles [1]. Current literature proves the hypothesis that melanocytes, the innate and adaptive branches of immunity, together with genetic polymorphisms, all synergize in the pathogenesis of vitiligo. Elevated serum titers of melanocyte-reactive antibodies against melanocytes are pathogenic in disease models both in vivo and in vitro and are assessed in individuals suffering from vitiligo [1]. Notably, the activity of vitiligo is not reflected by autoantibody titers [2]. In addition, vitiligo affects clearly demarcated areas and does not match with a widespread distribution mediated by autoantibodies; such observations lessen the pathogenic role of anti-melanocyte antibodies [3]. Conversely, research has shown that CD8+ T cells and increased IFN-γ play a key role and could provide potential therapeutic targets. Gene expression analysis of lesions revealed a predominant upregulation of IFNG gene [4,5] and genes related to IFNG induction, mainly chemokines CXCL9, 10, and 11, and their receptor CXCR3.

Histological analysis of active vitiligo lesions revealed an increase in the lymphocytic infiltrate in their periphery, mainly represented by CD8+ T lymphocytes [6]. Tyrosinase, Melan-A/MART-1, gp100, TRP-1, and TRP-2 are the proteins identified by studies on melanoma against which autoreactive CD8+ T lymphocytes are activated [7,8]. Studies performed ex vivo on autologous healthy skin explants demonstrate how CD8+ lymphocytes are necessary and sufficient for the destruction of melanocytes in vitiligo, unlike CD4+ cells, which were unable to do the same [9,10]. Similarly, effective responses following anticancer immunotherapy, with anti-PD1 agents that lift the inhibitor T-cell immune checkpoint, are associated with the activation of CD8+ T cells and their cellular infiltration into the tumor [11,12].

Additionally, various studies on murine models of vitiligo have demonstrated the correlation between an increased number of Treg cells and reduced severity of the disease. Moreover, it has been shown that injecting PD-L1-Fc into mice predisposed to vitiligo increased the concentration of Tregs in the skin and improved depigmentation. These findings suggest that the number of Treg cells is important for controlling the progression of vitiligo. Treg cells naturally reduce the proliferation and activation of autoreactive effector T cells, a phenomenon called anergy; phenotypic analysis of peripheral blood mononuclear cells suggests that melanocyte-reactive CD8+ T cells escape anergy in vitiligo patients [13]. Further studies will be needed to gain an in-depth knowledge on Treg cell deficits and their contribution to vitiligo; enhancing Treg cell function might provide novel avenues for drug discovery.

Vitiligo has long been an area of sorely unmet therapeutic need. However, new insights into the pathogenesis of vitiligo in recent years promise novel alternatives based on targeted molecules with high tolerability and response. Recent literature has clarified the implication of the IFN-γ pathway in the recruitment of T cells across initial and chronic disease stages. Small-molecular inhibitors against JAK are potent inhibitors of IFN-γ signaling and clinical trials using these drugs have provided promising results that anticipate their future approval as treatments for vitiligo. Disease recurrence after the discontinuation of these drugs depends on autoimmune tissue-resident memory cells, and interfering with IL-15 and their cutaneous retention might provide a durable treatment strategy. Future strategies for drug discovery should consider reestablishing the balance at the basis of immune regulation in the skin—which involves activating Treg cells or using factors that restore immune privilege in the hair follicle, stimulating the regeneration of melanocyte stem cells—which could replace laborious phototherapies and synergize with immunomodulating strategy therapies to develop improved combination treatments. Finally, in the management of drug-induced vitiligo, underlying therapy should be continued, and a tailored approached should be proposed to address the patient’s cosmetic impairment on a case-by-case basis. Topical corticosteroids or topical tacrolimus can be applied to the affected areas and, in selected cases, NB-UVB or medical camouflage therapy are appropriate.

### 2.2. Drug-Induced Vitiligo

#### 2.2.1. Leukoderma Related to Monoclonal Antibodies

**Figure 1:**
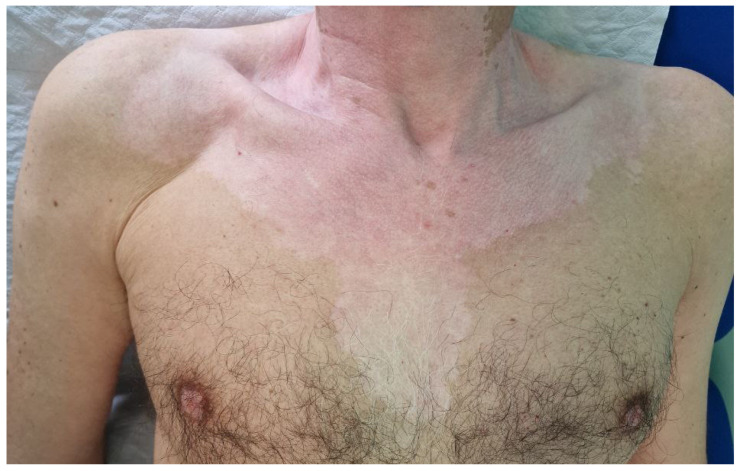
Vitiligo-like leukoderma. Acromic patches in a male subject receiving nivolumab for the treatment of classic Hodgkin’s lymphoma.

Hematological diseases involve inflammatory processes that can affect various body systems, including the skin. Medications, like immune checkpoint inhibitors (pembrolizumab, nivolumab, and ipilimumab), while enhancing immune responses against cancer cells, can also trigger autoimmune reactions, including vitiligo-like lesions due to immune dysregulation (Figure 1). Immune checkpoint inhibitors have been significantly associated with the occurrence of vitiligo [6,7] and the increase in vitiligo reports during treatment with monoclonal antibodies observed after 2010 was mainly related to the introduction of these drugs. The mechanism of vitiligo induced by pembrolizumab, nivolumab, and ipilimumab is thought to be immunological in origin and is based on the recognition of autoantigens present in the dermis/epidermis following the destruction of malignant cells [7]. 

However, pembrolizumab, nivolumab, and ipilimumab work by activating the immune system to target cancer cells. While they can lead to skin-related adverse effects, like vitiligo-like reactions, they do not directly target signaling pathways involved in skin pigmentation. For these reasons, it is plausible to attribute the mechanism of vitiligo induced by checkpoint inhibitors to both immune system response and the alteration in the expression of specific surface molecules triggered by these drugs.

#### 2.2.2. Hypomelanosis Related to Small-Molecule Inhibitors

Targeted therapy with BRAF and MEK inhibitors has been employed in patients with BRAFV600E-mutated multiple myeloma and hairy cell leukemia [14,15]. The onset of peculiar adverse events has been related to therapy and the cutaneous compartment is a main target organ: this is due to the specific actions of the inhibition of MAP kinase activation that is part of key pathways influencing cutaneous homeostasis [16,17]. The leukoderma induced by targeted therapies may be elucidated by the activation of antitumor cellular responses consequent to the blockade of target cells mediated by these molecular inhibitors; cell surface expression patterns and are reshaped by targeted therapies and these changes promote the activation of T cells specifically directed against the tumor. Clinically, symmetrical patches of leukoderma resembling vitiligo gradually appear over months [18]. The absence of Koebner’s phenomenon preference for sun-exposed areas, such as the face and hands, distinguish vitiligo-like reactions from actual vitiligo. 

Imatinib mesylate is a tyrosine kinase inhibitor used for the treatment of myeloid neoplasms. The drug is an effective blocker of the BCR-ABL fusion product in chronic myeloid leukemia and also of PDGF receptors and of KIT. The latter has a major role in melanocyte proliferation and function and its inhibition by the drug may explain hypomelanosis observed during treatment with imatinib. Subjects receiving this drug may show a progression of preexisting vitiligo or develop de novo hypopigmentation of the skin, which may be limited to the extremities or generalized. These manifestations are more evident in individuals with darker skin pigmentation and are usually reversible if treatment is discontinued [19]. The spectrum of adverse events concerning hypopigmentation related to systemic antineoplastic treatments is described in Table 1.

### 2.3. Hematologic Disorders Associated with Vitiligo 

In hematology, specific subtypes of cutaneous lymphoma may present with hypopigmented lesions, and certain genetically determined syndromes, such as ataxia teleangectasia, may be associated with vitiligo.

#### 2.3.1. Hypopigmented Mycosis Fungoides

Hypopigmented mycosis fungoides (HMF) is an extremely rare form of mycosis fungoides that presents with hypo/acromic patches that can be confused with vitiligo. Moreover, recent literature reported a case of HMF with granulomatous slack skin. HMF progresses slowly and presents with non-specific clinical features mainly characterized by multiple round or oval hypopigmented to achromic lesions on the trunk, limbs, buttocks, and groin [27]. In immunohistochemistry, the majority of HMFs are CD8+ T-cell lymphomas, while the epidermotropic infiltrate in classical MF is characterized by neoplastic CD4+ T cells. Considering that the simultaneous diagnosis of two rare variants in a single patient is exceptional, it is therefore recommended after the diagnosis of HMF to perform several biopsies on other suspicious lesions to support a definitive diagnosis.

#### 2.3.2. Ataxia Teleangectasia

Autosomal recessive syndrome ataxia teleangectasia (AT) is related to the genetic mutation of the ataxia teleangectasia mutated (ATM) gene. Patients are subject to a high incidence of cancer in general and of lymphoid malignancy in particular, which require constant and lifelong monitoring. Additionally, these individuals are predisposed to autoimmunity and are at risk of developing vitiligo [28,29].

## 3. Morphea

### 3.1. Overview of Morphea 

Morphea is the term indicating an inflammatory skin disorder that involves the dermis and may extend into the subcutaneous adipose tissue. Although cutaneous lesions are usually asymptomatic, morphea may cause significant morbidity owing to functional impairment and the risk of disfiguring sequelae.

**Figure 2:**
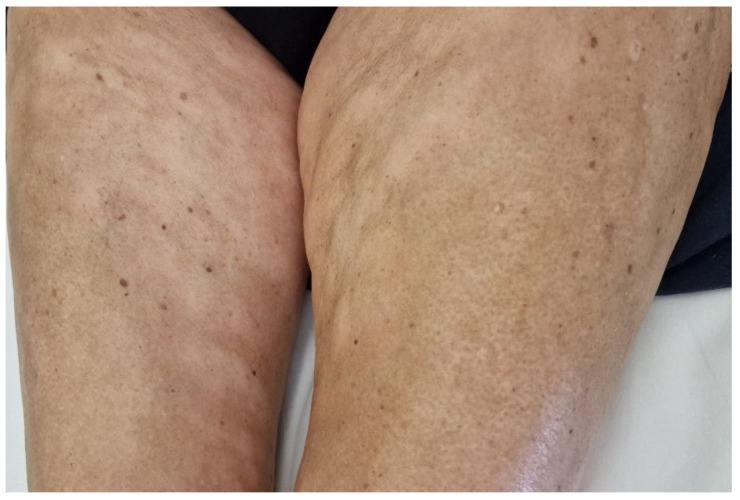
Plaque-type morphea. Hypopigmented plaques on the inner thighs of a female patient with monoclonal gammopathy.

The main subtype of this disorder, plaque-type morphea (Figure 2), presents with multiple lesions of variable size and asymmetric skin distribution—predominantly on the trunk—that develop as expanding plaques with central white induration surrounded by a peripheral “lilac ring”. Adnexal structures are usually destroyed in the sclerotic area, while a postinflammatory hyperpigmentation may replace the hypopigmentation in inactive lesions.

Activated T cells are recognized to have a major role in the pathogenesis of this group of disorders and may orchestrate key pathogenic mechanisms related to the production of vascular abnormalities and aberrant fibrogenesis. Specifically, the synthesis of extracellular matrix proteins, chiefly collagen, is strongly supported by cytokines derived from CD4+ Th2 cells. IL-4 and IL-13 increase the production of TGF-beta, which in turn activates fibroblasts to establish skin sclerosis [30,31]. Moreover, studies have associated the CXCL4 chemokine ligand to fibrosis in subjects with systemic sclerosis; CXCL4 is a potent promoter of Th2 responses and also has anti-angiogenic properties [32]. The pathogenic contribution of Th2 lymphocytes to skin fibrosis appears significant in the context of hematological malignancies on the basis that type 2 cytokines are involved in the progression of chronic lymphocytic leukemia, Hodgkin’s lymphoma, as well as certain other solid tumors. In this view, the targeted inhibition of key cytokines IL-4 and IL-13 might prove useful to improve symptoms of skin sclerosis, but this is still speculative [33]. 

Moreover, the role of oral retinoids has been investigated in patients with chronic GVHD, demonstrating a partial effect resulting from the interaction between these drugs and the TGF-beta pathway [34]. 

Imatinib also interferes with PDGF receptor signaling and was shown to contrast dermal sclerosis in animal models of systemic sclerosis [15]. This drug has also been assessed in chronic GVHD considering its effect on the profibrotic pathway mediated by the PDGF receptor [35]. Finally, Janus kinase inhibitors are established therapeutics both in myelodysplastic syndromes and in cutaneous immune mediated disorders, and are promising experimental agents for the treatment of cutaneous fibrosis [31].

### 3.2. Morphea and Its Association with Hematologic Disorders

Although a description of the range of clinical manifestations in the spectrum of morphea is beyond the scope of this review, certain variants are significant due to their association with hematological disorders. 

#### 3.2.1. Deep Morphea

Deep morphea features’ involvement beyond subcutaneous fat may clinically resemble eosinophilic fasciitis, a distinct fibrosing disorder with rapid clinical progression. The latter has been associated with myeloproliferative disorders and monoclonal gammopathy, as well as with aplastic anemia, thrombocytopenia, pancytopenia [36], and lymphoma [37,38]. While there are only anecdotal associations with deep morphea, for example with lymphoma, there is a certain overlap between the two conditions in the literature [39].

#### 3.2.2. Bullous Morphea

Bullous morphea refers to the development of cutaneous bullae in the context of substantial edema and lymphatic stasis. It is an uncommon presentation that may be seen in generalized morphea and morpheaform GVHD [40].

#### 3.2.3. Generalized Morphea

Generalized morphea may share cutaneous modifications that are similar to diffuse cutaneous systemic sclerosis; the trunk may become entirely affected and shows typical sparing of the periareolar circumference and the disease may extend along the extremities determining a puffy edema of the hands. The absence of internal organ involvement and of changes in the digits are sufficient to differentiate between the two disorders. Esophageal dysmotility, small intestine disfunction, interstitial lung disease, pulmonary arterial hypertension at risk for secondary heart failure, fibrotic cardiomyopathy, hypertension, and renal damage constitute the principal and debilitating internal organ affections related to systemic sclerosis, while they are not clinically relevant in generalized morphea. Finally, vascular changes are early events that are shared in the pathogenesis of both systemic sclerosis and morphea; however, digital sclerosis, Raynaud’s phenomenon, and cutaneous ulcers are features of systemic sclerosis alone [41]. 

### 3.3. Morpheaform Disorders

This is a group of inflammatory syndromes that are responsible for cutaneous sclerosis and that may mimic morphea (Table 2). When disease presents with asymmetric and focal involvement, this hints at possible local insults that may act as triggers [41]. Systemic sclerosis-like manifestations have been rarely reported in relation to certain cytotoxic antineoplastic drugs, such as bleomycin and taxanes [42,43,44,45], while a morpheaform presentation may be related to radiotherapy. Finally, cutaneous sclerotic changes related to neoplasia have been only rarely reported in the literature.

#### 3.3.1. Radiation-Induced Morphea

This is a delayed complication that may affect up to one in 500 subjects receiving radiotherapy and is preceded by irradiation many years earlier. In addition to the changes associated to radiation dermatitis, the clinical presentation is significant for sclerosis and dyspigmentation that affect the treated field and may extend further [46].

#### 3.3.2. Skin Sclerosis in Patients with Chronic Graft-Versus-Host Disease

Allogeneic hematopoietic stem cell transplantation (HSCT) is currently applied to a broad spectrum of conditions in hemato-oncology, ranging from lymphocytic tumors to myeloproliferative disorders, from myelodysplastic syndromes to aplastic anemia. Chronic graft-versus-host disease (GVHD) is a source of significant concern, due to the increase in the number of transplants in general and of those from matched, unrelated donors in particular, and constitutes a clinical challenge, especially in cases that are not well managed with first-line measures, relying on oral corticosteroids and skin-directed treatments. The clinical presentation and histology of chronic GVHD in subjects who received allogeneic HSCT may be overlapping with those of morphea, as well as of lichen sclerosus. Manifestations may also resemble eosinophilic fasciitis in certain cases. Moreover, chronic GVHD affecting the skin may present with hypopigmentation or vitiligo-like manifestations [52].

These clinical entities may be associated with the recruitment of eosinophils that reflects a reactive pattern to a defined immune stimulus paralleled by tissue and blood eosinophilia. Reported frequencies of sclerosis in chronic GVHD are variable, ranging from 13 to 53% of cases, and the risk factors are not defined; finally, total body irradiation is a commonly used conditioning strategy that may predispose to subsequent onset of skin sclerosis [53].

#### 3.3.3. Paraneoplastic Scleroderma-like Syndrome

Plasma cell proliferation disorders, a spectrum of conditions ranging from monoclonal gammopathies to overt multiple myeloma, have been associated in the literature with scleroderma-like manifestations. The pathogenesis is thought to be mediated by the direct infiltration of malignant cells in the skin, and cutaneous lesions are hyperpigmented rather that hypocromic [47].

Among subjects with hypereosinophilic syndrome, skin involvement is observed in half of cases. The myeloproliferative variant of hypereosinophilic syndrome has been reported in the literature with sclerotic changes in the skin and subcutaneous tissue [54]. 

Finally, in paraneoplastic scleroderma, the underlying neoplasm is thought to be the triggering factor; therefore, cutaneous changes usually subdue when the associated malignancy is treated successfully [55]. Additionally, paraneoplastic scleroderma has also been reported in cases of Kaposi sarcoma. Here, the pathogenesis could be mediated by profibrotic TGF-beta and VEGF hyperproduction by fibroblasts infected with HHV8 [48].

## 4. Syndromic Albinism

Albinism is a worldwide genetic condition resulting from mutations in at least 20 genes implicated in the production or transportation of melanin. It can affect the skin, hair, and/or eyes [56]. There are two main subtypes:

- Non-syndromic albinism, in which symptoms are related to impaired melanin biosynthesis. Individuals diagnosed with non-syndromic oculocutaneous albinism (OCA) typically exhibit ocular anomalies ocular anomalies, including nystagmus, optic nerves affections, and foveal hypoplasia [56].
- Syndromic form, which is characterized by various non-pigmentary symptoms [47].

Treatment focuses on symptom management and supportive care, including prophylaxis against infection and ultraviolet protection for the skin and eyes (applying topical sunscreen, wearing photoprotective clothing and sunglasses, and counseling with an ophthalmologist). HSCT is the only curative treatment for hemophagocytic lymphohistiocytosis (HLH) in Chediak–Higashi syndrome (CHS) and Griscelli syndrome type 2 (GS2) [57]. However, neurological complications persist and cannot be halted, especially in cases of CHS and Griscelli syndrome type 1 (GS1). Immunosuppressive therapy has been reported as an effective palliative treatment for GS2 until HSCT can be performed [58], while treatment with G-CSF can be useful to reduce frequency and severity of infections in MAPBP-interacting protein (MAPBPIP)-deficiency syndrome [59]. Finally, hypopigmentation does not normally improve.

### 4.1. Hematologic Conditions Linked with Syndromic Albinism

Patients affected by a syndromic form of albinism are vulnerable to pyogenic bacterial infections, especially in the respiratory tract and the skin, due to impaired neutrophil number and function [48].

Syndromic forms of albinism include CHS, subtypes 1–11 of Hermansky–Pudlak syndrome (HPS1-11), Griscelli syndrome types 1, 2, and 3, and MAPBPIP-deficiency syndrome [47,49].

Mutations in genes involved in the intracellular biogenesis and trafficking of lysosomes and lysosome-related organelles are responsible for these conditions [50]. More specifically, genetic dysfunction leads to aberrant lysosome-related organelles (LROs), such as melanosomes, granules in platelets and in T lymphocytes (CD8+), as well as in type II pneumocytes, causing the classic OCA phenotype [46,51].

Ocular symptoms typically manifest at birth in patients with HPS2 or CHS. MAPBPIP deficiency, on the other hand, is characterized by short stature, while the presence of HLH is common in CHS or GS2. Neurological complications are more prevalent in CHS compared to other forms of syndromic albinism. Finally, chronic neutropenia is generally observed in HPS2 and MAPBPIP-deficiency syndrome, while it is usually transient in CHS and GS2 [47].

Blood anomalies and their clinical significance are summarized in Table 3.

#### 4.1.1. Chediak–Higashi Syndrome 

CHS is a rare, autosomal recessive disorder characterized by multi-organ symptoms including hypopigmentation that varies from slight hypopigmentation to partial oculocutaneous albinism. It is also characterized by bleeding and hematologic abnormalities that can progress to HLH [60,61] and progressive neurological deterioration [62]. CHS results from mutations in the Lysosomal Trafficking Regulator (LYST) gene that causes impaired membrane dynamics and organelle fission [57].

Early-onset presentation occurs in 85% of individuals with CHS, resulting in the development of HLH during childhood, while the adolescent/adult variant exhibits a less severe phenotype and does not progress to HLH [57].

#### 4.1.2. Hermansky–Pudlak Syndrome 

HPS arises from disruptions in intra/extracellular transport systems. It presents with hypopigmentation, ocular abnormalities, and bleeding tendency; subtypes vary in manifestations, including pulmonary fibrosis, granulomatous colitis, and immunodeficiency [57]. 

Chemical analysis of hair reveals a reduced melanin concentration and a decreased eumelanin/pheomelanin ratio in HPS patients compared to control individuals. These alterations can contribute to increased oxidative damage mechanisms that are implicated in carcinogenesis and/or melanomagenesis pathways [63,64]. 

#### 4.1.3. Griscelli Syndrome 

Griscelli syndrome includes types 1, 2, and 3, each associated with specific genetic mutations and clinical outcomes.

GS1 patients are generally affected by neurological complications caused by mutations in the gene encoding the motor protein myosin-5a (MYO5A) [65]. GS2 is caused by mutations in the RAB27A gene, which encodes a small GTPase that regulates vesicular fusion and trafficking. Patients affected by GS2 present hematologic disturbances rather than neurologic problems since the gene RAB27A is absent from the nervous system [66]. The clinical characteristics of Griscelli syndrome type 3 (GS3) result instead from mutations in the melanophilin gene (MLPH) causing a reduced pigmentation of the skin and hair [66]. 

#### 4.1.4. MAPBP-Interacting Protein Deficiency

The deficiency of the late endosomal–lysosomal MEK binding partner 1 (MP1)-interacting protein (also known as p14 and MAPBPIP) causes a primary immunodeficiency syndrome resulting in neutropenia, partial albinism, short stature, and neurological abnormalities [67].

## 5. Conclusions and Future Perspectives

In conclusion, vitiligo, morphea, and albinism represent common manifestations that can not only be part of a dermatological disorder, but can either indicate underlying hematological pathologies or result from systemic therapies administered to treat such conditions. Vitiligo may arise in patients treated with checkpoint inhibitors or imatinib, or it may be associated with conditions like ataxia telangiectasia or mycosis fungoides. Morpheaform lesions can develop in patients receiving treatments like bleomycin or taxanes, post-radiotherapy, or as an expression of graft-versus-host disease (GVHD). They may also be associated with monoclonal gammopathies progressing to multiple myeloma or hypereosinophilic syndrome. Furthermore, in children presenting with oculocutaneous albinism, it is imperative to consider and exclude various forms of albinism, including CHS, HPS, GS, and MAPBPIP-deficiency syndrome. Hypopigmentation skin disorders could thus represent not only potential side effects of systemic therapies, but also important indicators for further investigation within the hematological spectrum. These aspects emphasize the necessity of a continuous dialog between hematologists and dermatologists in patient management. Identifying and understanding these dermatological manifestations are crucial for timely diagnosis, appropriate management, and improved patient outcomes in the context of hematological disorders. Pigmentary alterations constitute a potential cause of severe cosmetic concern in patients living with hematologic conditions; therefore, the treating dermatologist is a key figure in improving the overall quality of life and in promoting adherence to therapy. While novel targeted therapeutics that may be tolerable, even in patients receiving systemic cancer treatments, are highly anticipated, current management must also consider cosmetic camouflage as a strategy that may be employed by patients in their daily lives to address cosmetic impairments. Finally, although the onset of skin manifestations does not influence the natural history of underlying hematologic conditions, timely skin-directed treatment, as well as the option of replacing the responsible drug when feasible, has the potential to alter the progression of cutaneous involvement.

## Figures and Tables

**Table 1:** Systemic cancer treatments associated with vitiligo-like depigmentation and scleroderma.

Drug	Adverse Effect	Incidence	Proposed Mechanism	Mean Time to Onset (Months)
Immune checkpoint inhibitors (pembrolizumab, nivolumab, and ipilimumab) [20]	Vitiligo-like depigmentation	Common (11% in anti-CTLA-4 therapy; 25% in anti-PD-1 therapy)	The immune system recognizes self-antigens within the dermis/epidermis following the destruction of malignant cells	2
serine/threonine-protein kinase B-Raf (BRAF) inhibitors(vemurafenib, dabrafenib, and encorafenib) [21]	Vitiligo-like depigmentation	Rare (no data available regarding precise incidence)	Autoimmune destruction of melanocytes mediated by CD8+ T lymphocytes	8
Imatinib mesylate [22,23]	Vitiligo-like depigmentation	Rare (no data available regarding precise incidence, although an incidence rate of 23% has been reported in the clinical trial by Nishida et al.)	Inhibition of the tyrosine-protein kinase (KIT) receptor leads to apoptosis of melanocytes	NA
Bleomycin [24,25]	Scleroderma-like lesions	Rare (no data available regarding precise incidence)	Bleomycin induces an upregulation of type I procollagen synthesis and glycosaminoglycan production in the lungs and skin	6
Taxane-based agents(paclitaxel and docetaxel) [24,26]	Scleroderma-like lesions	Rare (no data available regarding precise incidence)	Paclitaxel and docetaxel lead to an increase in serum IL-6 (a pro-fibrotic cytokine) levels	13

Abbreviations: PD-1: programmed death 1; CTLA-4: cytotoxic T-lymphocyte-associated antigen 4; IL-6: interleukin 6; NA: not available.

**Table 2:** Scleroderma-like disorders and hypothesized etiological factors or triggers.

Scleroderma-Like Disorder	Hypothesized Etiological Factors/Triggers
Radiation-induced morphea [46]	Radiotherapy: radiation exposure typically precedes the onset of cutaneous sclerosis by several years
Paraneoplastic scleroderma-like syndrome [47,48]	Plasma cell proliferation disorders; cases associated with Kaposi sarcoma have been reported
Drug-induced systemic sclerosis-like syndrome [24]	Systemic cancer treatments, including taxanes and bleomycin
Morphea [49]	Viral or bacterial infections, such as by Borrelia burgdorferi, may serve as a trigger
Atrophoderma of Pasini and Pierini [50]	Viral or bacterial infections, such as by Borrelia burgdorferi, may serve as a trigger
Eosinophilic fasciitis [41]	It typically begins a few days following strenuous exercise;cases mediated by drugs (most notably statins) and cases associated with aplastic or hemolytic anemia, thrombocytopenia, myelodysplastic syndrome, and malignant lymphoproliferative disease have been reported
Chronic sclerodermoid GVHD [41]	GVHD, commonly observed following bone marrow and stem cell transplants, occurs as a result of immunocompetent T lymphocytes within the graft recognizing histocompatibility differences and attacking immunodeficient recipient tissue
Nephrogenic systemic fibrosis [51]	It occurs in renal insufficiency patients undergoing dialysis or kidney transplantation;also reported in acute kidney injury or stage IV-V chronic kidney disease patients receiving gadolinium
Toxic scleroderma-like syndromes [41]	Toxic-oil syndrome: aniline-denatured and refined rapeseed oil; Eosinophilia–myalgia syndrome: L-tryptophan;Vinyl-chloride disease: chronic inhalation of vinyl-chloride

Abbreviations: GVHD, graft-versus-host disease.

**Table 3:** Hematologic alterations and the related clinical implications in syndromic forms of albinism.

	CHS	GS2	HPS2	MAPBPIP-DS
Platelet abnormalities	Thrombocytopathy (platelets with dense granules)	none	Thrombocytopenia	none
Lymphocyte abnormalities	Alteration in T-cell and NK cytotoxic activity; occasionally large granules in lymphocytes	Alteration in T-cell and NK cytotoxic activity	Alteration in T-cell and NK cytotoxic activity	Defects in B-cell, T-cell, and NK cytotoxic activity
Neutrophil abnormalities	Transient neutropenia can be observed; large granules in PMN	Transient neutropenia can be observed	Neutropenia	Neutropenia
Evolution to HLH	yes	yes	yes	no

Abbreviations: CHS: Chediak–Higashi syndrome; GS: Griscelli syndrome; HPS: Hermansky–Pudlak syndrome; MAPBPIP-DS: MAPBPIP-deficiency syndrome; HLH: hemophagocytic lymphohistiocytosis; PMNs: polymorphonuclear neutrophils; NK: natural killer.

## Data Availability

Since this review does not present original data, there are no new datasets associated with this study. All information discussed in this review is based on previously published literature, and the relevant references are provided within the manuscript.
